# Geometric Reproducibility of Three-Dimensional Oral Implant Planning Based on Magnetic Resonance Imaging and Cone-Beam Computed Tomography

**DOI:** 10.3390/jcm10235546

**Published:** 2021-11-26

**Authors:** Franz Sebastian Schwindling, Sophia Boehm, Christopher Herpel, Dorothea Kronsteiner, Lorenz Vogel, Alexander Juerchott, Sabine Heiland, Martin Bendszus, Peter Rammelsberg, Tim Hilgenfeld

**Affiliations:** 1 Department of Prosthetic Dentistry, Heidelberg University Hospital, 69120 Heidelberg, Germany; sophia.boehm@med.uni-heidelberg.de (S.B.); christopher.herpel@med.uni-heidelberg.de (C.H.); mail@lorenz-vogel.de (L.V.); peter.rammelsberg@med.uni-heidelberg.de (P.R.); 2 Institute of Medical Biometry and Informatics, Heidelberg University Hospital, 69120 Heidelberg, Germany; kronsteiner@imbi.uni-heidelberg.de; 3 Department of Neuroradiology, Heidelberg University Hospital, 69120 Heidelberg, Germany; alexander.juerchott@med.uni-heidelberg.de (A.J.); sabine.heiland@med.uni-heidelberg.de (S.H.); martin.bendszus@med.uni-heidelberg.de (M.B.); tim.hilgenfeld@med.uni-heidelberg.de (T.H.)

**Keywords:** dimensional measurement accuracy, cone beam computed tomography, magnetic resonance imaging, imaging, dental implants, permanent dental restoration

## Abstract

This study aimed to investigate the geometric reproducibility of three-dimensional (3D) implant planning based on magnetic resonance imaging (MRI) and cone-beam computed tomography (CBCT). Four raters used a backward-planning approach based on CBCT imaging and standard software to position 41 implants in 27 patients. Implant planning was repeated, and the first and second plans were analyzed for geometric differences regarding implant tip, entry-level, and axis. The procedure was then repeated for MRI data of the same patients. Thus, 656 implant plans were available for analysis of intra-rater reproducibility. For both imaging modalities, the second-round 3D implant plans were re-evaluated regarding inter-rater reproducibility. Differences between the modalities were analyzed using paired t-tests. Intra- and inter-rater reproducibility were higher for CBCT than for MRI. Regarding intra-rater deviations, mean values for MRI were 1.7 ± 1.1 mm/1.5 ± 1.1 mm/5.5 ± 4.2° at implant tip/entry-level/axis. For CBCT, corresponding values were 1.3 ± 0.8 mm/1 ± 0.6 mm/4.5 ± 3.1°. Inter-rater comparisons revealed mean values of 2.2 ± 1.3 mm/1.7 ± 1 mm/7.5 ± 4.9° for MRI, and 1.7 ± 1 mm/1.2 ± 0.7 mm/6 ± 3.7° for CBCT. CBCT-based implant planning was more reproducible than MRI. Nevertheless, more research is needed to increase planning reproducibility—for both modalities—thereby standardizing 3D implant planning.

## 1. Introduction

Guided implant surgery based on three-dimensional (3D) cone-beam computed tomography (CBCT) imaging has become part of daily clinical routine. Several studies have addressed the accuracy of this workflow—more specifically its trueness and precision. In this context, trueness refers to the difference between a virtual implant plan and the definitive, actual position of the implant after surgery [1]. Trueness is in the range of 1–2 mm for the implant entry-level and tip, and around 5° for the implant axis [2,3,4,5,6,7]. In contrast, precision can be measured by repeating the insertion of one virtually planned implant multiple times, e.g., by using several phantom models. Precision is below 0.5 mm, 0.2 mm, and 2° for implant tip, entry-level, and angulation, respectively [1].

Less information is available regarding the accuracy of radiation-free guided implant surgery based on magnetic resonance imaging (MRI). The available evidence indicates that its trueness is inferior to that of the CBCT-based approach [8,9,10,11,12]. Several factors could account for this, including the increased susceptibility of MRI to artifacts [13,14], its lower spatial resolution, and distortions caused by the nonlinearity of its gradient. No data are currently available regarding the precision of MRI-based guided implant surgery.

A central consideration of the guided-implant-surgery workflow has not yet been addressed: the geometric reproducibility of the 3D implant-planning procedure. The 3D implant-planning procedure is a distinct part of the guided-implant-surgery workflow. It includes selection of the implant and its virtual positioning in relation to bony and dental structures, as part of a prosthetically driven backward-planning concept. The available studies on workflow accuracy (both trueness and precision) do not include analysis of the 3D implant-planning step; instead, they refer to comparisons further “downstream” in the workflow, either by comparing the virtually planned position with the definitive position (trueness), or by comparing definitive implant positions with each other (precision). This lack of evidence is inconsistent with the research consensus to investigate every step of the digital workflow [15]. Importantly, 3D implant planning has surgical and prosthetic implications. Consequently, planning reproducibility encompasses more than radiologic studies focusing on the imaging data itself, e.g., on the repeatability of linear or volumetric measurements [16,17,18].

Because 3D implant planning is subject to complex underlying considerations, it must be assumed that geometric deviations will occur during repeated planning. Nevertheless, implant positioning has concrete biomechanical consequences: Depending on the clinical situation and type of restoration, even minor increases in inclination compared with the angle of a perfectly inserted implant can place the peri-implant bone under significantly greater strain [19,20,21]. This could compromise implant survival by adding detrimental stress, similar to what was reported for strain by loosening and tightening the implant healing screw [22]. Interestingly, the combination of MRI and x-ray-based imaging could be promising to further investigation of bone regeneration in this context [23].

If, however, raters are clearly instructed on the principles of where to place implants, then imaging data might be the determining factor in the geometric reproducibility of 3D implant planning. In the present study, 3D implant planning was performed by trained raters who had received thorough planning instructions. All raters planned implants with the same software, using the same patient cases imaged with both CBCT and MRI. This enabled the geometric reproducibility of CBCT- and MRI-based guided implant surgery to be quantified and compared in vivo. The aim of this study was to determine whether reproducibility is higher for CBCT-based or MRI-based implant planning. The hypothesis was that CBCT-based planning is more reproducible.

## 2. Materials and Methods

### 2.1. Study Design

Twenty-seven patients in need of 41 implants were included in a prospective clinical trial (approval number S-404/2014, registration: German Clinical Trials Register, DRKS00014239; title: Planning of dental implants with a new imaging modality). Fifteen patients required one implant, 10 patients two implants, and two patients three implants. Twenty-four implants were placed in the mandible, and 17 in the maxilla. Preoperative MRI and CBCT scans were acquired only a few days apart. The following participant inclusion criteria were defined: reconstruction of tooth gaps or free-end situations; restoration with fixed or removable implant concepts; at least three remaining teeth distributed in more than one quadrant to allow image registration; and a minimum interval of three months between tooth extraction and implantation. Exclusion criteria were need for a two-stage surgical procedure with separate bone augmentation and implant insertion; contraindications to 3 Tesla (T) MRI; age below 18 years; pregnancy; and claustrophobia. This study was conducted in accordance with the Declaration of Helsinki. Written informed consent was obtained from all study participants. We declare an overlap between the participants in this and previous studies [9].

### 2.2. Imaging Data

Dental MRI examinations were performed preoperatively with a 3T MRI system (Magnetom Tim Trio, Siemens Healthineers; Erlangen, Germany) using a dedicated 15-channel dental coil (Mandibula, Noras MRI products GmbH; Höchberg, Germany), as described elsewhere [14]. A multi-slab acquisition with view-angle tilting sampling perfection with application-optimized contrasts using different flip-angle evolution prototype sequence (MSVAT-SPACE) was used. Sequence parameters were repetition time 1170 ms; echo time 6.4 ms; field of view 168 × 131 mm^2^; voxel size 0.4 × 0.4 × 0.4 mm^3^; matrix 384 × 300 × 80; slice oversampling 220%; 80 slices; 10 min total examination time including planning and patient positioning. Preoperative CBCT scans were performed as part of clinical routine, using the following acquisition protocol: 3D Accuitomo 170, J Morita; field of view, 8 × 8 cm^2^; tube voltage, 90 kV; tube current, 7 mA; 14 bit; 360° rotation in 17 s; 560 frames; isotropic voxel size of 160 µm.

### 2.3. Virtual Implant Positioning

Virtual implant positioning was performed using standard software (coDiagnostiX; Dental Wings Inc.; Montréal, QC, Canada). DICOM data (MRI, CBCT) and previously digitized stone casts of the participants’ clinical preoperative situations were imported into the software. Then, four dentists with an average of four years’ experience in dental imaging were introduced to the key principles of implant planning [24]. In addition, the following requirements were defined: use of bone-level implants for anterior regions and tissue-level implants for posterior regions; distance of 1.5 mm to the adjacent teeth and 3 mm to adjacent implants; and minimum distance of 1.5 mm to the inferior alveolar nerve. Simultaneous bone-augmentation procedures—sinus elevation in posterior maxillary regions, bone spreading, and bone splitting—were permitted. For training purposes, ten illustrative cases were selected from the dataset, and the rationale for each implant plan was discussed in detail. One month later, the raters performed implant planning for all 41 implant cases in both modalities, which were presented anonymously in random order. The implants were then virtually planned for a second time, with a time lag of two weeks.

### 2.4. Geometric Deviation Analyses

**Figure 1:**
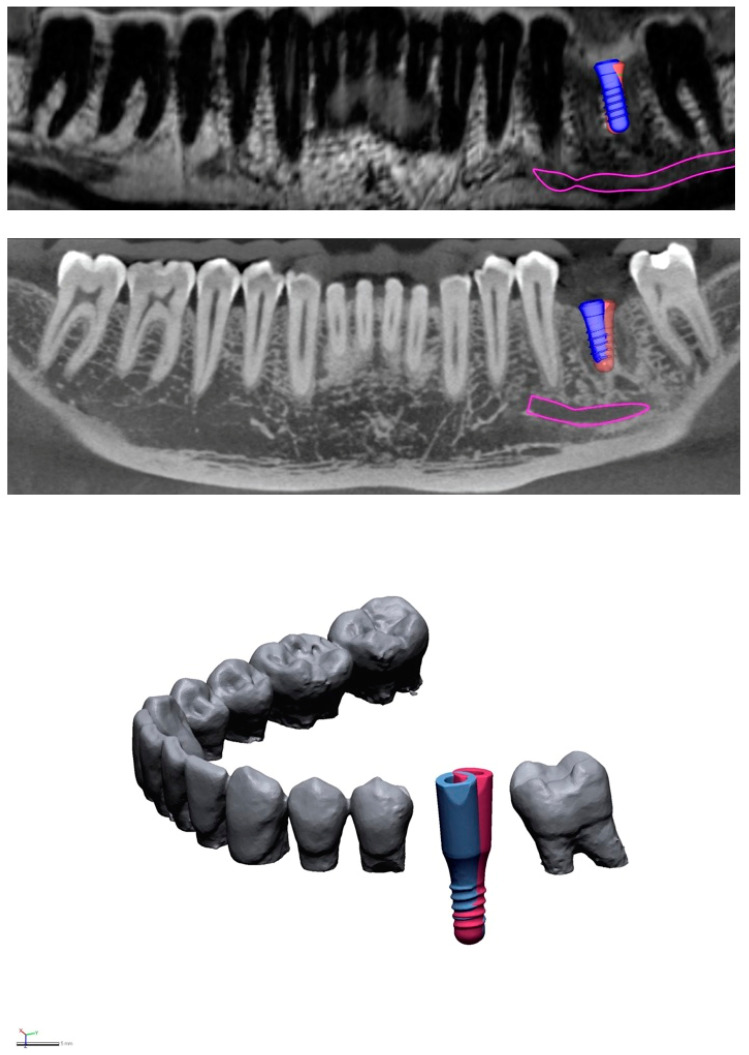
Illustrative slices from MRI (**top**) and CBCT (**middle**) scans. Virtual implant placement was performed twice in each imaging modality with a time lag in between to prevent learning bias. The slices show superimposition of the two plans in area 36 (blue implant: first implant plan, red implant: second implant plan). Deviations can be seen between the two plans regarding implant tip, entry-level, and axis. Pink: nerve canal (CBCT) and alveolar bundle (MRI). Comparison of the two images reveals that image quality is superior in CBCT, due to its higher resolution and lower susceptibility to motion artifacts. (**Bottom**): The virtual plans of both modalities were exported from the 3D planning software and imported into reverse-engineering software. Using this software, geometric deviations were evaluated at the implant tip, entry-level, and axis.

To evaluate the reproducibility of 3D implant planning, all implant plans were exported from the planning software as STL files. To assess intra-rater reproducibility (Figure 1), the first and second plan (Figure 1) for each implant were imported into reverse-engineering software (Geomagic DesignX, 3D Systems; Ettlingen, Germany), for both imaging modalities. When exporting two implant plans of one specific patient case from the coDiagnostiX software, they are saved within an identical global coordinate system. This means there is no need for subsequent alignment, and the superimposition error equals 0 µm for this step. As a next step, however, virtual implants were imported and aligned—which was associated with an alignment error. The aligned, virtual implants were of the same type and size as the implants planned in the specific site, and they were equipped with scan bodies, as described in previous studies [3,25,26]. For alignment, the scan bodies within the exported plans were used as the static reference data, while the virtual implants were moved. The Geomagic DesignX-tool “scan to scan alignment” was used, and three different points were selected for the procedure. To quantify the error of aligning, the dimensional congruence between the virtual implants and the exported implant plans was measured at ten random implant cases. Root mean square was utilized to indicate the magnitude of deviations from zero between the two datasets. A high root mean square value indicates a low-degree of 3D matching accuracy in superimposed files (tool: “measure mesh deviations”). The maximum/minimum deviation values were arranged to be +500/−500 μm. The deviation between the virtual implants and exported implant plans was 29 ± 3 µm. In the context of expected outcome differences in the 1000-µm-range, an alignment error of below 30 µm can be regarded as minor, even when doubling the error (the alignment is performed twice when comparing two implant plans).

Geometric intra-rater deviations at the implant tip, entry-level, and axis were measured for each implant in both modalities separately, as described elsewhere [3,25]. The descriptive analysis included mean values, standard deviations (SD), and boxplots. Because of the paired structure of the data (i.e., MRI and CBCT measurements were available for every patient), statistical analysis was performed using paired t-tests. The significance level was set to 0.05. To assess inter-rater reproducibility, deviations at the tip, entry-level, and axis of each implant were compared among all six combinations of raters, for each modality separately. To exclude inter-round learning bias from the analysis, only the second-round implant plans were evaluated for CBCT and MRI. Based on a total of four raters, six inter-rater combinations are possible. Here, each rater’s data are used three times for analysis: Rater 1 data are compared with Rater 2 data, and then again with Rater 3 data, and then again with Rater 4 data. To account for this three-fold use, a Bonferroni *p*-value correction was performed [27]. Because of the explorative nature of the study, all *p*-values are solely descriptive in nature.

## 3. Results

### 3.1. Intra-Rater Reproducibility: Geometric Deviations between First and Second 3D Implant Plans

**Figure 2:**
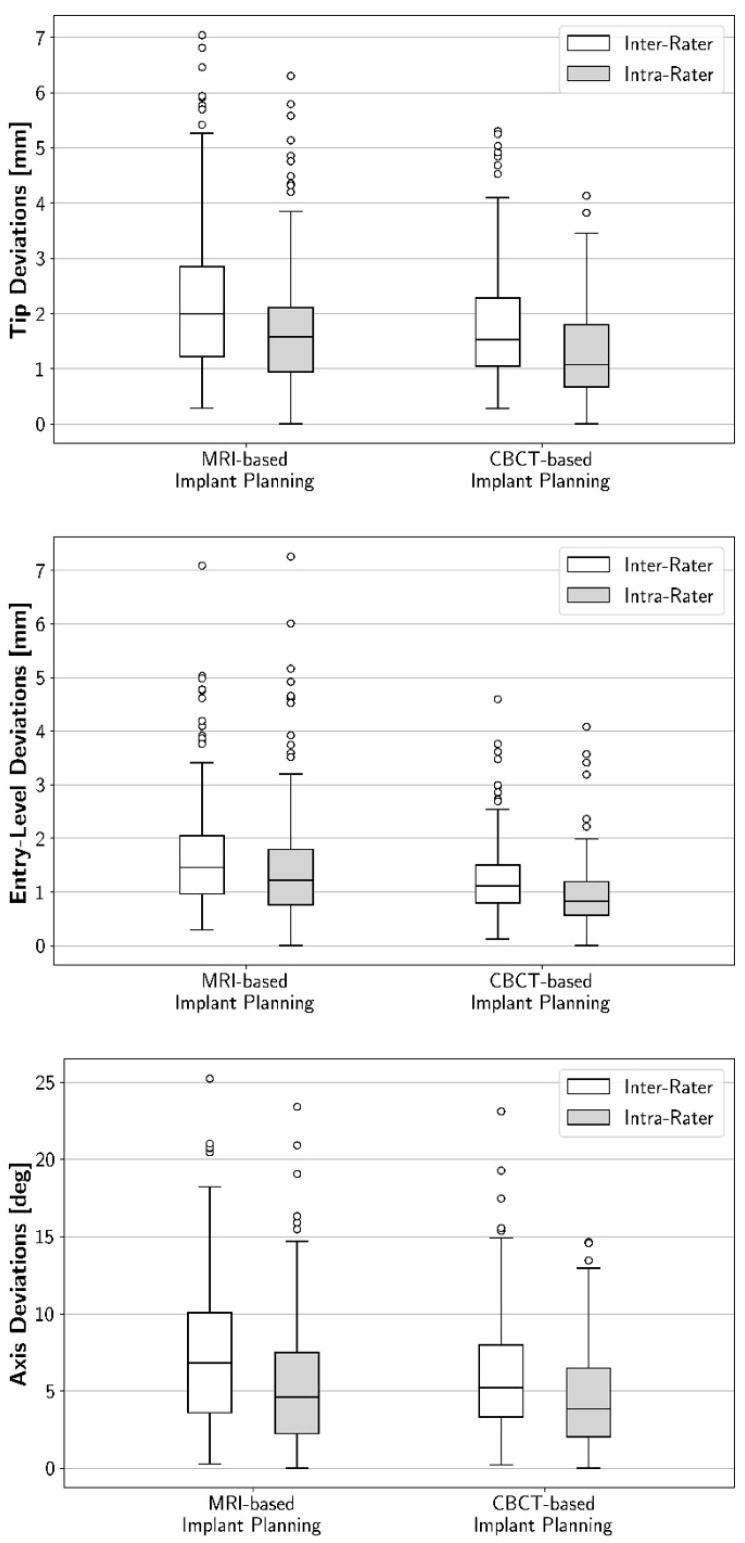
Boxplot diagrams illustrating, for MRI and CBCT, intra-rater and inter-rater deviations for implant tip, entry-level, and axis. For all three areas of the implant, intra-rater reproducibility and inter-rater reproducibility were higher for CBCT-based implant plans than for MRI-based ones. Outliers are depicted as hollow circles.

Intra-rater reproducibility for the implant tip, entry-level, and angulation was lower for MRI than for CBCT (Figure 1). Regarding the implant tip, the mean intra-rater deviation was 1.7 mm (SD: 1.1 mm) for MRI, and 1.3 mm (SD: 0.8 mm) for CBCT (Figure 2). The mean intra-rater deviation for the implant entry-level was 1.5 mm (SD: 1.1 mm) for MRI, and 1 mm (SD: 0.6 mm) for CBCT. Regarding implant angulation, the mean intra-rater deviation was 5.5° (SD: 4.2°) for MRI, and 4.5° (SD: 3.1°) for CBCT. The two modalities were also compared for each individual rater separately. Significant differences were observed between CBCT- and MRI-based deviations for the implant entry-level (except for one rater) and, in part, for the implant tip (Table 1).

### 3.2. Inter-Rater Reproducibility: Geometric Deviations between the Raters’ 3D Implant Plans

Inter-rater reproducibility for the implant tip, entry-level, and angulation was also lower for MRI than for CBCT. Regarding the implant tip, the mean inter-rater deviation was 2.2 mm (SD: 1.3 mm) for MRI, and 1.7 mm (SD: 1 mm) for CBCT (Figure 2). The mean intra-rater deviation for the implant entry-level was 1.7 mm (SD: 1 mm) for MRI, and 1.2 mm (SD: 0.7 mm) for CBCT. Regarding implant angulation, the mean intra-rater deviation was 7.5° (SD: 4.9°) for MRI, and 6° (SD: 3.7°) for CBCT. A separate evaluation of the various inter-rater combinations was performed. After Bonferroni-correction, significant differences between the modalities were detected for only three of the six rater combinations (Table 2).

## 4. Discussion

The present study is the first to provide comprehensive reproducibility data for 3D implant planning based on CBCT and MRI. A large number of implant plans were analyzed: each rater planned 41 implants twice in each imaging modality, resulting in a dataset of 656 implant plans to measure intra-rater reproducibility. For the analysis of inter-rater reproducibility, only the second round of plans was selected (328 plans), and all possible 1986 comparisons between the four raters were analyzed.

Our findings confirm the hypothesis that CBCT-based 3D implant planning is more reproducible than MRI-based planning. Reproducibility is important when evaluating the quality of a diagnostic procedure. Although several studies have investigated the geometric accuracy of other, subsequent parts of the guided-implant-surgery workflow [2,3,4,5,6,7], analysis of planning reproducibility has so far been neglected. Studies on the reproducibility of linear bone measurements are not sufficient in this context, because 3D implant planning involves more than the performance of single linear measurements. On the contrary, the procedure requires complex decision-making based on several measurements, the available bone, prosthetic and surgical demands, implant region, as well as the number and position of residual teeth. The reproducibility of 3D implant planning is a meaningful parameter for an in-depth characterization of implant planning quality. This is for two reasons. First, for the first time, our findings allow deviations within the 3D implant-planning procedure to be compared to inaccuracies further “downstream” in the workflow. Second, they allow direct comparison of the performance of two imaging modalities (CBCT and MRI) when aiming to achieve the optimal implant position.

The reproducibility of CBCT-based 3D implant planning can be put into context with the accuracy (more specifically: trueness) of the guided-implant-surgery workflow further “downstream.” Studies have reported trueness of approximately 1.1 mm for the implant entry-level, 1.4 mm for the implant tip, and 3.6° for the implant axis [2,28,29,30,31]. In the present study, the reproducibility of CBCT-based 3D implant planning was in the range of 1.1 mm, 1.5 mm, and 5.3° for implant tip, entry-level, and angulation, respectively, when averaging the values for intra- and inter-rater reproducibility. Our data therefore illustrate that planning reproducibility deviates by approximately the same extent as all subsequent steps of the workflow combined. Given the number and type of subsequent steps (3D printing of the surgical guide, bonding of metal inserts, repeated positioning of the guide during osteotomy, implant insertion), this result was unexpected. It remains unclear why research has thus far focused on assessing the accuracy of subsequent fabrication steps, instead of attempting to standardize the 3D planning procedure. Reproducibility could be optimized by enhancing software properties and using artificial intelligence guidance during the 3D implant-planning procedure. Specific examples could include automatic measurements of tooth gaps or bone height/width, and automatic detection of the axes of teeth adjacent to the implant site, which would enable automated alignment of implant axes. These tools might help clinicians to standardize planning decisions, making the workflow faster and more predictable, and thereby increasing cost-efficiency.

Comparison of the two modalities revealed that MRI-based implant plans were less reproducible than CBCT-based plans in terms of intra- and inter-rater reproducibility. The study was designed to reduce potential biasing factors such as operator inexperience (related to implant planning as well as use of MRI) by providing raters with thorough training and clear instructions. Therefore, other factors might explain the inferior performance of MRI. The most important factor in this context might be image quality. The higher spatial resolution of CBCT along with its lower vulnerability to motion artifacts (due to a shorter acquisition time) results in superior visualization of intraoral hard tissue. This enables clearer delineation of the implant target zone, i.e., bone quantity and distance to neighboring structures in the area of the future implant. However, it must be emphasized that the reproducibility of MRI-based implant planning was unexpectedly high, considering its lower resolution (isotropic MRI voxel size of 440 µm vs. 160 µm in CBCT, equating to a 21-fold lower resolution factor for MRI) and longer acquisition time (7.45 min vs. 17 s, 27-fold increase). Despite these inferior properties, mean deviations in geometric reproducibility were only 1.33-fold larger in MRI, The absolute differences between MRI and CBCT for implant entry-level/impIant tip/implant axis were only 0.4 mm/0.5 mm/1° (at an intra-rater level). We consider that the difference in planning reproducibility between the two modalities as clinically insignificant in the context of a CBCT-based workflow accuracy, which shows a standard deviation of 0.34 mm/0.44 mm/1.42° [32].

This study has several limitations. First, the findings are based on optical measurements. Implant positions were exported from the 3D implant-planning software and analyzed using best-fit alignment in reverse-engineering software. Thus, alignment errors might have biased the results. Second, the MRI technology applied requires dedicated coils and sequences with artifact suppression. Not every radiology department has access to this technology, thus limiting the wider applicability of the results. Third, the relatively good performance of MRI compared with CBCT remains surprising, because there was a structural bias in favor of CBCT-based implant planning. This bias relates to the fact that reference bodies were present in the CBCT scans, unlike in the MRI scans. The presence of the metal references was due to the study design, which—because of ethical reasons—required the verification of an MRI-based implant plan before surgery. Although the raters were not given any details regarding the reference body, it was probably used for orientation during implant planning, thus standardizing CBCT-based planning and disadvantaging MRI-based planning. Finally, because of the small sample size, the clustering of more than one implant in patients could not be considered during statistical analysis.

## 5. Conclusions

The intra- and inter-rater reproducibility of 3D implant planning based on CBCT imaging was higher than that of MRI-based planning. Both intra-rater and inter-rater deviations between virtual implant plans based on CBCT are in the range of deviations associated with the trueness of the complete workflow. More research is therefore required to increase reproducibility, in order to standardize and automate 3D implant planning.

## Figures and Tables

**Table 1:** Geometric deviations between the two imaging modalities (CBCT and MRI) were compared for each rater separately, for the implant tip, entry-level, and axis. Significant differences are marked in bold. * To avoid artificially increasing the study power, no statistical analysis of mean values was performed.

Parameter	Rater	Mean Difference between Modalities	95% Confidence Interval	*p*-Value
Tip [mm]	Mean	0.5	(0.3, 0.7)	*
	1	0.6	(0.1, 1)	**0.015**
	2	0.4	(0, 0.9)	0.061
	3	0.6	(0.3, 1)	**0.002**
	4	0.3	(0, 0.7)	0.090
Entry-level [mm]	Mean	0.5	(0.3, 0.7)	*
	1	0.6	(0.3, 1)	**0.002**
	2	0.7	(0.3, 1.2)	**0.003**
	3	0.6	(0.2, 1)	**0.002**
	4	0.2	(−0.2, 0.6)	0.283
Axis [°]	Mean	1	(0.2, 1.8)	*
	1	1.4	(−0.3, 3.2)	0.109
	2	0.2	(−1.8, 2.2)	0.826
	3	0.8	(−0.3, 1.9)	0.135
	4	1.5	(0.1, 2.9)	**0.037**

**Table 2:** Geometric deviations between the two imaging modalities (CBCT and MRI) were compared for all possible inter-rater combinations separately, for the implant tip, entry-level, and axis. Significant differences are marked in bold. * To avoid artificially increasing the study power, no statistical analysis of mean values was performed.

**Parameter**	**Rater Combination**	**Mean Difference between Modalities**	**95% Confidence Interval**	***p*** **-Value** **(Bonferroni-Corrected)**
Tip [mm]	Mean	0.5	(0.3, 0.7)	*
	1 vs. 2	0.4	(−0.1, 1)	**0.033**
	1 vs. 3	0.2	(−0.3, 0.7)	0.084
	1 vs. 4	0.6	(0.1, 1.1)	**<0.001**
	2 vs. 3	0.2	(−0.2, 0.6)	0.399
	2 vs. 4	0.6	(0.1, 1)	0.669
	3 vs. 4	1.0	(0.5, 1.5)	1
Entry-level [mm]	Mean	0.4	(0.3, 0.6)	*
	1 vs. 2	0.5	(0, 0.9)	**0.030**
	1 vs. 3	0.3	(−0.1, 0.7)	0.156
	1 vs. 4	0.4	(0, 0.8)	**<0.001**
	2 vs. 3	0.4	(0.1, 0.6)	0.147
	2 vs. 4	0.5	(0.1, 0.8)	0.060
	3 vs. 4	0.7	(0.3, 1)	0.399
Axis [°]	Mean	1.4	(0.7, 2.2)	*
	1 vs. 2	1.2	(−0.8, 3.2)	0.102
	1 vs. 3	1.0	(−0.6, 2.6)	**0.012**
	1 vs. 4	2.8	(1, 4.6)	**0.039**
	2 vs. 3	−0.1	(−2.1, 1.8)	0.720
	2 vs. 4	1.7	(0.1, 3.3)	1
	3 vs. 4	2.2	(0.5, 3.9)	0.675

## Data Availability

Due to ethical reasons, data cannot be provided.
